# Progress in Isolation and Molecular Profiling of Small Extracellular Vesicles via Bead-Assisted Platforms

**DOI:** 10.3390/bios13070688

**Published:** 2023-06-28

**Authors:** Daria Kozhevnikova, Vasiliy Chernyshev, Alexey Yashchenok

**Affiliations:** 1 Skoltech Center for Photonic Science and Engineering, Skolkovo Institute of Science and Technology Skolkovo Innovation Center, 121205 Moscow, Russia; daria.kozhevnikova@skoltech.ru; 2 National Medical Research Center for Obstetrics, Gynecology and Perinatology Named after Academician V.I. Kulakov, 117997 Moscow, Russia; vchernys@gmail.com

**Keywords:** small extracellular vesicles, exosomes, beads, isolation, molecular characterization, liquid biopsy

## Abstract

Tremendous interest in research of small extracellular vesicles (sEVs) is driven by the participation of vesicles in a number of biological processes in the human body. Being released by almost all cells of the body, sEVs present in complex bodily fluids form the so-called intercellular communication network. The isolation and profiling of individual fractions of sEVs secreted by pathological cells are significant in revealing their physiological functions and clinical importance. Traditional methods for isolation and purification of sEVs from bodily fluids are facing a number of challenges, such as low yield, presence of contaminants, long-term operation and high costs, which restrict their routine practical applications. Methods providing a high yield of sEVs with a low content of impurities are actively developing. Bead-assisted platforms are very effective for trapping sEVs with high recovery yield and sufficient purity for further molecular profiling. Here, we review recent advances in the enrichment of sEVs via bead-assisted platforms emphasizing the type of binding sEVs to the bead surface, sort of capture and target ligands and isolation performance. Further, we discuss integration-based technologies for the capture and detection of sEVs as well as future research directions in this field.

## 1. Introduction

To date, numerous methods have been developed, tested, and compared to isolate and purify small extracellular vesicles (sEVs) for fundamental research as well as research and development of new technologies for disease diagnostics and new drug carriers for therapeutics [1,2,3,4]. Although no single standard protocol exists, the most commonly used approach that is considered to be a gold standard for sEV isolation from biological samples is differential centrifugation, which is purely based on the size and density that affect sedimentation rate at a given centripetal force [5,6]. The final step of differential centrifugation protocol is ultracentrifugation (UC), which allows obtaining a sEVs pellet at the most common force of 100,000 g [7,8]. Further purification can be achieved by additional ultracentrifugation steps [9,10,11]. Other ultracentrifugation methods are based on sucrose or iodixanol to form a density gradient allowing to separate and purify sEVs based on their buoyant density and can be coupled with UC [2,9]. Size-exclusion chromatography [12], polymer-based precipitation [13,14], field-flow fractionation [15], ultrafiltration [16,17], asymmetric depth-filtration [18], immuno-affinity capture [19] and different combinations of such methods [2,9,17,20] have been tested to improve the yield and purity of obtained sEVs samples and to find an optimal isolation method, which also depends on the end goal. Isolation of sEVs becomes especially difficult when working with more complex biological fluids such as human whole blood, plasma, and serum due to the presence of lipoproteins, such as very-low-density lipoproteins (VLDLs), low-density lipoprotein (LDLs), high-density lipoprotein (HDLs), albumin and other proteins and their aggregates, the removal of which is still an obstacle primarily due to the overlap of physical properties between sEVs and such contaminants [21]. The absence of a single solution to completely remove such proteins during sEV isolation produced a broad range of reported sEVs concentrations in blood [14,18,22]. Moreover, microvesicles (MBs) and apoptotic bodies (ABs) present in biological fluids also contributed to the heterogeneity of the samples (Table 1). MVs originated from the outward budding of the plasma membrane and exerted similar biological functions to that of sEVs. ABs are formed during cell apoptosis when cytoskeleton fragmentation causes the plasma membrane to swell outward.

Immuno-affinity capture in the form of stand-alone particles or integrated technologies is the proposed solution to such problems, which allows isolating subpopulations of sEVs with minimal contamination. Although a limited number of subpopulations can be isolated in a short period of laboratory time [23], such methodology received attention in recent years because it allows directly quantifying sEV subpopulations according to their membrane markers and is compatible with further sEV analysis, such as PCR or mass spectrometry (MS).

In this review, multiple approaches that are based on the use of sEV membrane composition for the isolation of sEVs are presented and discussed, highlighting their advantages and limitations. We focus on bead-assisted platforms, which rely on catching sEVs by surface-modified beads made of different materials, such as polymers, silica, magnetically responsive microparticles and others. We also highlight the area of integrated technology that combines bead-assisted approaches along with microfluidic technologies for harvesting and quantification of sEVs.

## 2. Outline of sEV Isolation Using Bead-Assisted Platforms

The isolation of sEVs using bead-assisted platforms consists of the following steps: (i) mixing the beads with sEVs; (ii) incubation of the mixture; (iii) separation of the beads with adsorbed sEVs from the unbounded sEVs; and (iv) analysis of the molecular composition of isolated sEVs.

Cell culture media and different bodily fluids, such as blood plasma, urine, lymph and others, are the primary sources of sEVs. Prior to blending sEVs with the beads, the sEVs samples are pre-isolated with ultracentrifugation, size-exclusion chromatography, ultrafiltration or a combination thereof to separate sEVs from larger size vesicles, cell debris, cells and plasma proteins. The analysis of the literature showed that the main pre-cleared method is ultracentrifugation, which includes three main steps: low-speed centrifugation during a short time period, high-speed centrifugation with an extended time period to remove cell debris and microvesicles, and ultra-high speed centrifugation or ultracentrifugation (UC) at forces of 100,000× *g* or greater to precipitate exosomes. At the same time, size-exclusion chromatography and PEG precipitation are also used to purify sEVs samples and reduce the cost and time of sample preparation.

After mixing the beads and sEVs, the mixture is incubated at room temperature for a defined time period that depends on a specific protocol.

The third step includes the separation of sEVs bound to the surface of the beads from unreacted sEVs and other impurities that remain in a working solution. Depending on the type of the beads (magnetic and nonmagnetic), either magnet or centrifugation can be applied to collect the beads with bound sEVs.

At the final stage, whether membrane receptors or interior molecules of sEVs are investigated, different methods are applied for the analysis of the molecular content of sEVs directly on the beads or in the free state after they are released from the bead surface.

## 3. Nonmagnetic Beads

**Figure 1:**
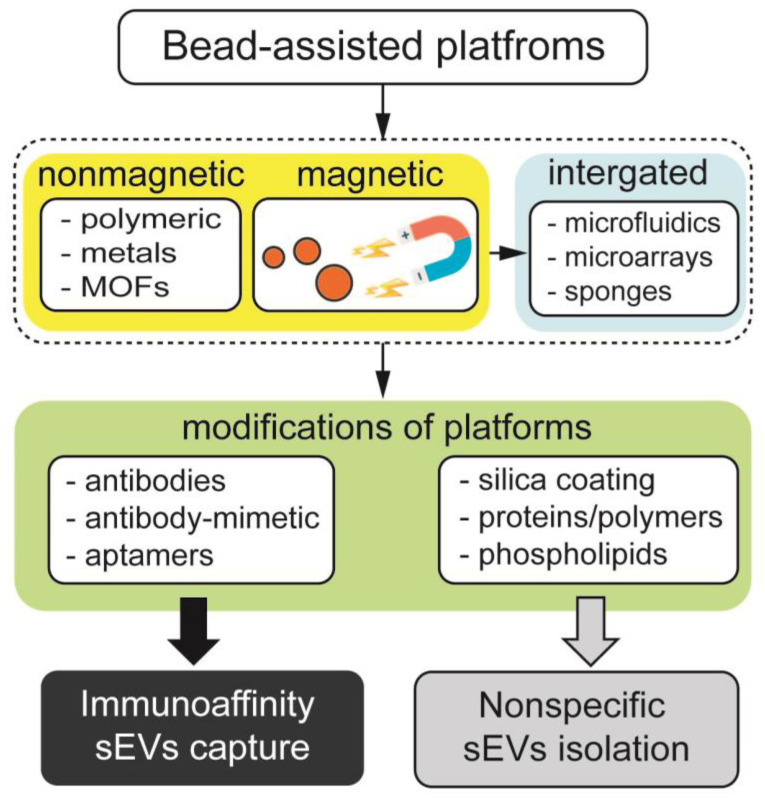
Classification of bead-assisted platforms based on nonmagnetic particles, magnetically responsive beads and those integrated with microfluidics, microarrays and porous materials. Middle row depicts the functionalization of bead-assisted platforms by coatings and organic molecules, leading to nonspecific and immunoaffinity enrichment of sEVs.

The use of polymeric materials for sEV isolation was commonly used and presented in the past years. Two types of approaches exist and are based on specific or nonspecific initial sEVs capture (Figure 1). Specific sEV isolation is based on the use of polymeric particles containing capture ligands, such as antibodies, aptamers, peptides and others that can specifically interact with target molecules on the membrane of sEVs. This allows isolating a subpopulation of sEVs from the pool of sEVs and other impurities present in the sample in the first step, followed by additional sample processing such as labeling of the capture ligands with tag-conjugated antibodies/aptamers for analysis or RNA isolation. Nonspecific sEVs capture is based on the passive adsorption of sEVs to the particle surface in the initial step, followed by sEVs labeling with tag-conjugated antibodies/aptamers for further analysis. A subpopulation cannot be obtained for further analysis after nonspecific capture. For the convenience of the readers, we have summarized the most effective bead-assisted platforms for the isolation, characterization and quantification of sEVs in Table 2.

### 3.1. Immunospecific sEVs Capture

One of the most commonly used affinity-based isolation techniques is based on the coupling of particles (e.g., polymeric materials) with bioaffinity ligands (e.g., antibodies or antibody mimetic) for immunoaffinity capture of a subpopulation of sEVs containing the chosen membrane proteins followed by further characterization of sEVs.

Katsu et al. isolated CD171+ neuron-specific sEVs that were later freed from bound anti-CD171 antibody–resin conjugates by lowering pH with glycine-HCl [24]. Flow cytometry was used to confirm the isolation of sEVs that contain neuron-specific markers, SNAP25 and synaptophysin, as well as common sEVs markers, CD81 and CD63. The obtained subpopulation of sEVs was eventually used for RNA extraction and miRNA expression analysis. The group was able to show that miRNA from neuro-derived sEVs in plasma can represent miRNA alterations in the brain and be used as biomarkers of amyotrophic lateral sclerosis. Yin et al. immobilized CD3-targeting aptamers in the shape of a caliper on the gold nanoparticles (AuNP) surface by forming an Au-S linkage to obtain and distinguish T-cell CD3 monomeric and dimeric sEVs subpopulations [25]. To isolate the CD3+ subpopulation of sEVs, the Au–caliper was mixed with sEVs to obtain the Au–caliper–EVs complex. sEVs bound to affinity oligonucleotide probes were released by successively incubating Au–caliper–sEVs complex with complementary oligonucleotides that are more thermodynamically preferable on the backbone stand of the caliper compared with affinity oligonucleotide probes. The same steps were used for the plasma of a skin transplantation mouse model, which showed the applicability of such a method for the isolation of CD3+ sEVs directly from biological fluids. The sEVs released from the Au–caliper–sEVs complex were further analyzed by nano-flow cytometry using FITC anti-CD3 antibody, which confirmed that the sEV membrane protein is intact. The obtained sEV subpopulations were eventually used for small RNA isolation and miRNA sequencing. Immunoaffinity isolation of sEVs based on the use of non-magnetic beads was also applied to plant studies [26,27]. Beads coupled to antibodies against markers such as Tetraspanin 8 (TET8) were used to isolate and purify specific subpopulations of plant sEVs for further analysis.

**Figure 2:**
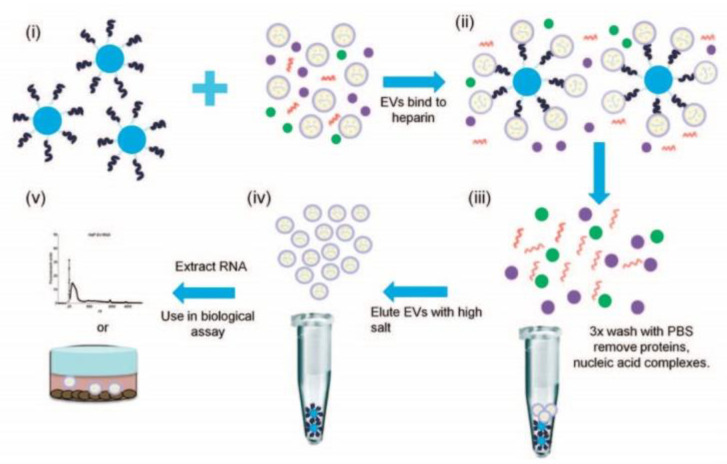
Workflow of isolation and purification of small extracellular vesicles using heparin-coated agarose beads. (**i**) The beads are incubated with sEVs derived from different cell lines, (**ii**) formation of a sEVs/bead complex, (**iii**) removing proteins and nucleic acids by washing with PBS, (**iv**) release of sEVs from the beads using a concentrated salt solution and (**v**) extraction of RNA from sEVs for analysis. Reprinted with permissions from reference [29]. Copyright 2015, Springer Nature.

In addition to specific protein markers presented on the surface of sEVs, research groups used other interactions between sEVs and beads [28]. Commercial heparin affinity chromatography beads (Affi-Gel^®^ Heparin Gel) were mixed with a cell culture medium of 293 T-cell line that was concentrated by 100 kDa filter prior to sEV isolation and, after overnight incubation, were centrifuged to remove proteins and nucleic acid complexes (Figure 2) [29]. sEVs were later unbound from the beads with high salt (NaCl) into the supernatant, and sEV-free beads were concentrated in the form of a pellet by centrifugation. The obtained sEVs from the supernatant were applicable for further analysis and RNA extraction. Pan W. et al. developed an EV-FISHER platform that is constructed from zirconium-based metal–organic frameworks and PO4(3−)-spacer-DNA-cholesterol (PSDC) [30]. The group showed that EVs could be effectively isolated from cell-culture media (MDA-MB-231) and plasma of breast cancer patients by their interaction with cholesterol present on the EV-FISHER and purified by low-speed centrifugation (12,800 g). It was also shown that DNase I can be used after isolation to detach EVs for further analysis, e.g., nano-flow cytometry and mass-spectrometry, as it hydrolyses the DNA in PSDC and allows for obtaining pure EVs. The amount of EVs isolated from breast cancer patients and healthy donors by EV-FISHED that contained the GPC-1 marker was shown to correlate with the clinical stage of the patient and reflect therapeutic efficacy based on nano-flow cytometry results.

**Table 2:** Application of bead-assisted platforms along with integrated technologies for isolation, characterization and quantification of sEVs.

Isolation Platform	Capture Ligand	Targeting Ligand	EV Source	Performance	Reference
AuNPs	Anti-CD3 aptamer	CD3	Cell culture; plasma of a skin transplantation mouse model	Capture capacity 22 μg/mg	[25]
Metal–organic framework (EV-FISHER)	Cholesterol	Glypican-1	Cell culture; breast cancer plasma	Capture efficiency 74.2%	[30]
Aldehyde microbeads	-	EpCAM and HER2	Cell culture; breast cancer serum	Purity ≈ 5 × 10^9^ particles µg^−1^ of protein	[31]
Magnetic beads	Anti-CD63, anti-CD9 antibodies	CD63 and CD9	Cell culture; human plasma or serum	Capture efficiency 75–80%	[32]
	Anti-CD63 antibody	CD63 microRNA	Plasma of high-risk cardiovascular disease patients	Capture efficiency 74%, miRNA extraction 91%	[33]
	Anti-CD63, anti-MUC1 aptamers	CD63 and MUC1	Cell culture; breast cancer plasma	Capture efficiency ~ 60%, release efficiency ~ 20%	[34]
	Tim4 protein	Phosphatidylserine	Cell culture; mouse serum, human urine	78.1% of total peptides	[35]
	Combination of hydrophilic and lipophilic groups	-	Urine cancer patients	Capture efficiency 95%	[36]
	PEG-assisted	-	Cell culture; human plasma	Capture efficiency ~40 × 10^10^ particles/mL; purity 19.2 × 10^10^ particles per mg protein	[37]
	Ti(IV) ions, phospholipid derivative 1,2-distearoyl-sn-glycero-3-phosphorylethanolamine	CD9 and CD63	Prostate cancer human urine	Capture efficiency > 81%	[38]
	Anion-exchange coating	CD81 and HSP70	Cell culture; human plasma	Capture efficiency >90% purity ~ 4×10^11^ particles per mg protein	[39]
Integrated microfluidic (ExoSearch)	Anti-CD9, anti-CD81, anti-CD63 antibodies anti-α-IGF-1R, anti-EpCAM, anti-CA125, anti-CD9, anti-CD81 and anti-CD63 antibodies	CA125, EpCAM, HE4 and CD24 IGF-1R/p-IGR-1R	Ovarian cancer human plasma ovarian and non-small-cell lung cancer human plasma	Capture efficiency ~79.7%, binding capacity ~ 5 µg, biotinylated antibody per mg beads; LOD 0.281 pg/mL for IGF-1R, 0.383 pg/mL for p-IGF-1R	[40,41]
Integrated microfluidic device	Anti-EpCAM antibody	-	Cell culture; breast cancer human plasma	Sensitivity 90%, specificity > 95%	[42]
ExoCounter	Anti-CD9 antibody	CD9, CD63, CD147, CEA and HER2	Cell culture; colorectal, lung, breast and ovarian cancer human serum; glaucoma or interstitial lung disease/pulmonary fibrosis	LOD 1.16 ng protein for cell culture; 0.39 μg for serum	[43]
Integrated magnetic-electrochemical exosome (iMEX)	Anti-CD63 antibody	EpCAM, CD24, CA125, HER2, MUC18 and EGFR	Cell culture; ovarian cancer human plasma before/after treatment	LOD 3 × 10^4^ vesicles	[44]
Electric field-induced release and measurement (EFIRM)	Anti-CD63 antibody	GAPDH mRNA	Cell culture; mice serum and saliva after injection of H460 cells	Capture efficiency ~ 85%	[45]
Miniaturized micronuclear magnetic resonance (μNMR) system	Anti-CD235a antibody	CD235a, CD55, CD47 and CD44	Packed red blood cell unit	LOD ∼ 2 × 10^6^ MV/μL with dynamic range up to ∼2 × 10^8^ MV/μL	[46]

### 3.2. Nonspecific sEVs Capture and Labeling

Other groups performed quantification of immunolabeled sEVs by first allowing them to passively adsorb to the bead surface and then labeling them with primary and secondary antibodies for analysis by using techniques such as flow-cytometry, which is commonly referred to as microbead-assisted flow cytometry. Such methods often require sEVs to be purified to minimize the effect of competitive nonspecific binding of contaminants to the bead surface.

Li et al. first allowed sEVs isolated by UC from MCF10a, MDA-MB-468, MCF-7 and SK-BR-3 cell culture media and human serum obtained from breast cancer patients and healthy donors to nonspecifically adsorb to aldehyde/sulfate latex beads [31]. After that, the bead–sEVs complexes were stained with anti-EpCAM or anti-HER2 primary antibodies and secondary antibodies for flow cytometry. Flow cytometry results for sEVs obtained from cell culture media were compared and agreed with Western blot. Based on the obtained results, it was found that both EpCAM+ and HER2 + sEVs can be effective diagnostic markers of breast cancer. Yang et al. pre-isolated sEVs from human plasma and cell culture media of 1617 PDAC cells by size-exclusion chromatography (SEC) and UC, respectively. The derived sEVs were biotinylated with EZ-link NHS-PEG4-Biotin, followed by collecting them with streptavidin-coated polystyrene particles and performing antibody staining for flow cytometry [47]. The streptavidin-coated latex beads were also compared with standard aldehyde/sulfate latex beads prior to immunolabeling. The streptavidin-coated particles showed a better signal compared to latex beads for the chosen sEVs markers (EpCAM, EGFR, MUC1 and PDAC that comprises an antibody cocktail, which included EpCAM, EGFR, MUC1, WNT-2 and GPC1 that were tested). The method was validated by using sEVs that were pre-isolated by SEC from the plasma of pancreatic ductal adenocarcinoma patients and healthy controls. In addition to the markers used for cell-culture media, the group also performed an analysis of additional markers (CD73, TIMP1, EphA2, LRG1 and Mesothelin). Flow cytometry results indicated that the multi-marker approach with the marker group consisting of EpCAM, EGFR, MUC1, WNT-2 and GPC1 allows to achieve high sensitivity and reproducibility. The described assay requires <4 h for analysis of 48 samples and LOD for sEVs protein and particle counts were estimated to be 41.3 ng and 1.3 × 10^7^ particles.

## 4. Magnetically Responsive Beads

An alternative to nonmagnetic particles, magnetic beads are yet another platform for trapping sEVs (Figure 1). Similar to nonmagnetic beads, magnetically responsive particles can carry bioaffinity ligands, which can specifically target sEVs surface receptors. Alternatively, magnetic beads functionalized with polymers, polysaccharides, phospholipids, etc., can capture sEVs through electrostatic interaction, hydrogen bonding or in an ion-dependent way. Unlike non-magnetic particles, magnetic beads can be collected using a permanent magnet by placing the magnet in a test tube containing an aqueous solution of beads and an analyte. Collecting magnetic beads with a magnet is advantageous over collecting non-magnetic beads with centrifugation because it can speed up the isolation of sEVs. In addition, due to the soft nature of the magnetic field generated by the magnets, the aggregation of beads and the adsorption of impurities can be minimal. Thus, the use of magnets to collect magnetic beads in isolation of sEVs can improve the purity of sEVs samples. In this section, we give an overview of the application of magnetic beads for the isolation of sEVs from cell culture media and bodily fluids in both specific and nonspecific manner.

### 4.1. Immunomagnetic sEV Enrichment

**Figure 3:**
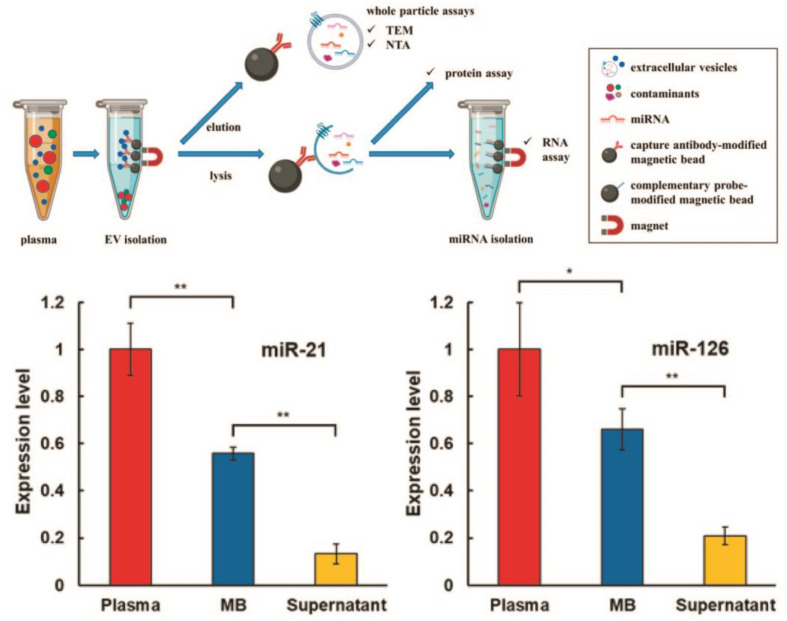
Workflow of two-step magnetic bead-based (2MBB) method for isolation of a subset of sEVs and sEV microRNAs. Anti-CD63 antibody-coated magnetic beads are first added to human plasma samples to capture sEVs. The isolated sEVs are then eluted for size and concentration analysis and lysed for analyzing proteins and RNAs. miRNAs are extracted using oligonucleotide-conjugated magnetic beads (**upper panel**). Levels of miRNAs hsa-miR-21-5p (**left**) and hsa-miR-126-3p (**right**) extracted from platelet-poor plasma, sEVs captured on magnetic beads and supernatant after magnetic beads relative to the spike-in exogenous cel-miR-238-3p (**bottom panel**). (** *p* < 0.01, * *p* < 0.05, Student’s *t* test). Reprinted with permission from reference [33]. Copyright 2020, PLOS ONE.

We start this section with an immunomagnetic-based approach, which relies on the selective targeting of sEV membrane receptors with ligand-functionalized magnetic beads. The antibodies targeting ligands are widespread for the separation of sEVs from both cell culture media and bodily fluids. The antibodies against human MHC Class II receptors were used to isolate sEVs from conditioned cell media of antigen-presenting cells [48]. In another example, antibody-coated paramagnetic beads allowed the collection of HER2-positive tumor sEVs (Tu-sEVs) of high purity from malignant ascites, which contain EVs secreted from various types of cells such as tumor cells, lymphoid cells and mesothelial cells [49]. Anti-CD9 and anti-PSMA functionalized antibodies magnetic beads were used to isolate sEVs from prostate cancer cell lines (LNCaP and PC-3), blood plasma of healthy volunteers and prostate cancer patients [50]. The CD-63 receptor was detected only in sEVs captured by anti-CD9 beads. The higher CD9 expression was identified in sEVs isolated from advanced cancer patients and from patients taking treatment but not in sEVs harvested from healthy volunteers and patients without metastasis. An untouched isolation strategy that specifically collected tumor-derived extracellular vesicles (T-EVs) from tumor tissue, preserving their native characteristics, was developed by Yu and co-workers [51]. The natural T-sEVs secreted by tumor tissues of cancer patients were isolated by a collection of undesired T-sEVs from the samples using magnetic beads carrying antibodies against surface receptors of sEVs characteristic of immune, endothelial and tumor cells. The avidity of vesicle antibodies immobilized on magnetic beads was increased by DNA linker, which allows not only isolating of sEVs from HEK-293 cell culture media and healthy human blood plasma volunteers but also a facile release of harvested EVs by enzymatically cleaving DNA linkers with DNase I [52]. A comparison of ultracentrifugation (UC-Exos), OptiPrepTM density-based separation (DG-Exos) and immunoaffinity capture using anti-EpCAM antibodies-coated magnetic beads (IAC-Exos) was performed by Tauro and co-workers [19]. It was found that IAC-Exos is the most effective method to isolate sEVs, which enabled the identification of cancer-related proteins in sEVs for the first time. Additionally, several cancer-related proteins were identified in IAC-Exos and components involved in Wnt and Ras signaling. In another study, Brett et al. compared immunomagnetic isolation techniques and commercially available kits, such as ExoQuick-TCTM (EQ, System Biosciences, Inc., Palo Alto, CA, USA), ExoSpinTM (Cell Guidance Systems, LLC., Cambridge, UK), and Total Exosome (Life Technologies, Inc., Carlsbad, CA, USA) for the isolation of sEV fraction from prostate cancer patient plasma [53]. The authors concluded that commercial sEV isolation kits are not able to eliminate a fraction of plasma proteins, while an immunoaffinity-based approach is highly effective in isolating sEVs of high purity. A high-resolution atomic force microscope analysis of single sEVs isolated by ultracentrifugation (UC) method and immunoaffinity (IA) approach using antibody-coated magnetic beads revealed that IA sEVs had higher surface roughness and bimodal size population compared to UC sEVs [54]. Zarovni et al. performed a comparison of commercially available (Thermo Scientific) and in-house developed immunoaffinity beads (HansaBioMed, Tallinn, Estonia) for sEV separation from complex and “crude” samples [32]. It was shown that immunoaffinity beads integrated with ELISA and PCR methods made it possible to reduce plasma volume to 0.1 mL for on-line analysis of proteins and mRNAs/miRNAs of sEVs. A two-step magnetic bead-based (2MBB) method for isolation of a subset of sEVs and sEVs microRNAs was designed by S. Chen and co-workers [33]. The first set of magnetic beads with anti-CD63 capture antibodies recognize the corresponding sEVs surface receptor, while the second set of magnetic beads functionalized with complementary oligonucleotides is specific to sEVs-associated microRNAs (Figure 3). The efficiencies of the 2MBB method amounted to 74% of sEVs enrichment and 91% of miRNA extraction compared with supernatant with spiked-in exogenous cel-miR-238 molecules.

Aptamers are yet another ligand for selective isolation of sEVs which in most cases surpass the affinity of antibodies due to their highly specific interaction with the target molecule, high affinity and small size. Zhang et al. demonstrated the isolation of sEVs with high purity from cell culture media by anti-CD63 aptamer-coated magnetic beads [34]. The subsequent release of captured sEVs is mediated by adding complementary sequences, which break the secondary structure of aptamer and nondestructively liberate sEVs from the bead surface. The trials on clinical samples showed a substantial difference in the number of captured sEVs between healthy volunteers and cancer patients using MUC1 aptamer instead of CD63. Then, working with clinical samples, the capture and release efficiencies of the assay were found to be about 60% and 20%, respectively.

Highly purified EVs were derived from cell-conditioned media, mouse serum and human urine by magnetic beads coated with T-cell immunoglobulin domain and mucin domain-containing protein 4 (Tim4), which strongly react with phosphatidylserine in a Ca^2+^-dependent manner [35]. The release of intact sEVs from magnetic beads was realized by the addition of an elution buffer containing the chelating agent EDTA (Ca^2+^ chelator). The Tim4-affinity purification method showed much purer fractions of sEVs compared with conventional ultracentrifugation and Total Exosome Isolation (TEI) reagent.

### 4.2. Nonspecific sEVs Trapping

In this section, we summarize research describing a nonspecific strategy for the isolation of sEVs by magnetically responsive beads. This strategy relies on different interactions between sEVs and the surface of magnetic beads, such as electrostatic, hydrophobic, lipophilic, etc. Although a nonspecific approach does not enable the isolation of a subpopulation of sEVs, as in the case of immunoaffinity, this strategy is costly and attractive in clinical implementation for genome and proteome analysis of sEVs.

A novel extracellular vesicles total recovery and purification (EVTRAP) method, which is based on the capture of sEVs by magnetic beads modified with a combination of hydrophilic and lipophilic groups, was developed by Wu and co-workers [36]. In combination with Western blot and liquid chromatography-mass spectrometry (LC-MS), the EVTRAP method showed superior capture efficiency of sEVs with high recovery yield (95%) and with about 2000 unique proteins from 0.2 mL of urine samples with a total LC-MS analysis of 90 min. The phosphoproteome analysis of urine sEVs demonstrated more than 860 phosphoproteins in 10 mL of urine versus 104 phosphoproteins in sEVs isolated by ultracentrifugation. The sEVs from cell culture media and from the blood plasma of healthy volunteers were successfully extracted by the MagExo approach operating in defined polyethylene glycol PEG concentrations [37]. One-step MagExo showed several times higher yield of the isolated sEVs than those of ExoQuick Ultra, UC and SEC, while purity was better than that achieved with ExoQuick Ultra. Two-step MagExo had a similar yield as one-step MagExo and comparable purity to SEC and UC. Sun et al. have extended the previous protocol ExtraPEG [55] to MagPEG (Magnetic beads and PEG-based protocol), which combines PEG precipitation and magnetic beads to isolate high-purity sEVs from conditioned cell media and human plasma [56]. The yield, purity and protein markers of sEVs derived by MagPEG were similar to that of the ExtraPEG method. MagPEG workflow combined with an automated liquid handling instrument allowed the isolation of up to 96 sEV samples from 5 μL pre-cleared serum in 45 min. J. Chen et al. have introduced the anion-exchange (AE) method for the isolation of sEVs from cell culture media and from plasma using anion-exchange magnetic beads [39]. The AE-based method enabled to reduce total isolation time to 30 min with high recovery yield (more than 90%) and improved purity of sEVs (several times higher compared with ultracentrifugation and AE chromatography). Bifunctional magnetic beads (BiMBs) functionalized with Ti(IV) ions and a phospholipid derivative, 1,2-distearoyl-sn-glycero-3-phosphorylethanolamine (DSPE), were developed for efficient enrichment of sEVs [38]. As stated by the authors, DSPE on BiMBs surface can penetrate sEVs membrane, while Ti(IV) ions bind to phosphate groups; thus, BiMBs can improve affinity and specificity for isolation and further molecular analysis of sEVs. The BiMBs showed the best isolation efficiency from both PBS and urine samples compared with magnetic beads immobilized with Ti(IV) only (TiMBs), DSPE only (DspeMBs) and ultracentrifugation (US). The proteome of isolated sEVs coupled with liquid chromatography-mass spectrometry resulted in identifying 3302 unique proteins with 95% of 100 top sEVs proteins in sEVs isolated using BiMBs that were higher compared with sEVs captured by TiMBs, DspeMBs and US. Additionally, sEVs captured with BiMBs have lower contaminants than sEVs harvested by other beads and US. Furthermore, phosphoproteomic analysis of prostate cancer sEVs from urine samples isolated by BiMBs with the help of trapped ion mobility spectrometry revealed 120 phosphoproteins with multiple positional isomers in phosphorylation. Magnetic beads coated with polysaccharide chitosan are capable of mediating sEV isolation from conditioned cell culture media (CCM) [57]. Magnetic beads at a final concentration of 0.5 mg/mL captured sEVs had characteristic sEVs markers, such as HSC70, CD63, CD9 and FLOT1. With increasing concentrations of chitosan-coated magnetic beads, the increase in the signal of sEVs markers was observed. In contrast, dextran-coated magnetic beads did not have any specific sEVs markers in the isolated material.

### 4.3. Quantification of Magnetically Isolated sEVs

Along with the development of sEVs isolation methods, techniques for the quantification of sEVs are also evolving. sEVs are first trapped on magnetically responsive particulate platforms, while the amount of sEVs is then determined by different detection methods [58,59,60,61]. The fluorescence-based approaches are most widely used for sEVs quantification. F. He et al. have proposed two methods for the quantification of sEVs. The first is based on DNA hybridization chain reaction (HCR) measurement for signal amplification using a bivalent-cholesterol-labeled DNA probe spontaneously inserted into the membrane of sEVs [62]. In the second method, sEVs are quantified by fluorescent copper nanoparticles forming in the presence of poly(thymine) as a result of the dissolution of anti-CD63 aptamer-functionalized copper oxide nanoparticles anchored to sEVs [63]. Both methods showed a low quantification limit (10^3^–10^4^ vesicles per μL) of determined sEVs when working with cell culture media and human serum of cancer patients. A new method for competitive detection of sEVs was developed by Yu and co-workers [64]. In this method, aptamer-functionalized magnetic beads specific to the CD63 protein of sEVs are hybridized with a Cy3-labeled short-sequence oligonucleotide probe complementary to a certain region of the aptamer. After adding sEVs to the magnetic bead–Cy3 probe complex, sEVs competitively bind to the aptamer-conjugated magnetic bead, resulting in a decrease in the fluorescence signal. Thus, the number of EVs can be estimated by measuring fluorescence intensity. A fluorescence-based platform for the sensitive detection of sEVs was developed by L. Huang and co-workers [65]. A three-step protocol was used for sEVs estimation. First, leukemia-derived sEVs were harvested by anti-CD63 antibody-modified magnetic beads. Then, a DNA primer comprising a nucleolin-recognition aptamer was applied to initiate a rolling circle amplification (RCA) reaction generating many repeat sequences. In the final step, gold nanoparticles were injected into the samples to induce the release of FAM dye. The fluorescence signal was continuously accumulated due to the transformation of FAM from the quenching state to the emission state. The sEVs in PBS and in spiked serum samples were successfully quantified with the limit of detection of 1 × 10^2^ vesicles per μL. A comparative study for the identification of EpCAM and HER2 surface proteins in the sEVs derived from MCF7, SKOV3, MDA-MB-231 and CHO cell lines and blood plasma of a healthy donor was carried out using anti-EpCAM and anti-HER2 designed ankyrin repeat proteins (DARPins) [66]. Both DRAPins showed high specificity to EpCAM and HER2 receptors, whereas the highly reactive nature DARPins, along with magnetic capture, allowed reducing total assay time. The performance of the bead-flow cytometry method for quantification of the molecular cargo in sEVs isolated from cell culture media and plasma of cancer patients was investigated in the following works [67,68]. The authors emphasized the following critical points for analysis of sEVs by bead-flow cytometry: (i) the right selection and preparation of beads and antibodies for sEVs capture; (ii) finding the optimal ratio of beads/antibody/sEVs; and (ii) the right isotype control to achieve the best separation of the isotype signal from the detection antibody signal. Additionally, gating on sEVs–bead complexes is yet another critical factor that should be set in the detection system to avoid analysis of sEVs–bead complexes aggregates. Following these criteria, one can achieve reliable and reproducible analysis of the molecular composition of sEVs using bead-flow cytometry method.

Apart from fluorescence-based quantification methods, a biocatalytic color-changing system based on horseradish peroxidase-encapsulated DNA flowers (HRP-DFs) was introduced by R. Zeng et al. for the quantitative screening of sEVs [69]. In this immunoassay design, target sEVs are bound to cholesterol-modified DNA probes-conjugated magnetic beads and CD63 aptamer-encoded HRP-DFs forming sandwich immune complexes. The complex is then magnetically separated to further initiate oxidation of 2,2′-azino-bis(3-ethyl-benzothiazoline-6-sulphonic acid) (ABTS), which undergoes a color change in the presence of H_2_O_2_. Thus, the color change of the ABTS-H_2_O_2_ system can be monitored by ultraviolet-visible spectrometry and is proportional to the concentration of sEVs.

**Figure 4:**
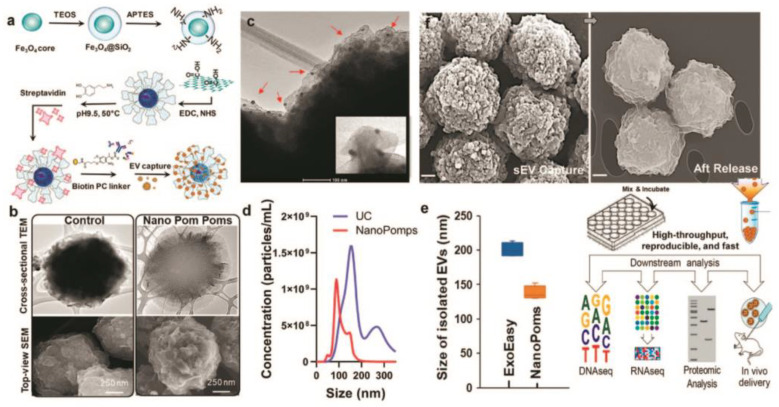
Schematic of the fabrication of Nano pom-poms capture platform for trapping of sEV and their release via on-demand photo-cleavage (**a**). TEM and SEM images of Nano pom-poms and a commercial nanoplatform (**b**). TEM image of captured sEVs on the surface of Nano pom-poms. Red arrows indicate captured sEVs with bound 10 nm antiCD63 gold nanoparticles (**c**). Nanoparticle tracking analysis of sEVs isolated by Nano pom-poms and ultracentrifugation (**d**). Nanoparticle tracking analysis of the size of sEVs isolated with Nano pom-poms and ExoEasy kit (**e**). SEM images of Nano pom-poms with captured sEVs and those after the release of sEVs via on-demand photo-cleavage. Workflow of multi-omic analysis and in vivo study (**f**). Reprinted with permission from reference [70]. Copyright 2022, Springer Nature.

Photo-click chemistry for specific marker capture and release of intact sEVs with the help of 3D-structured nanographene magnetic particles (NanoPoms) with unique flower pom-poms morphology was developed by N. He and co-workers [70]. The results showed improved specificity and sensitivity for detecting urological tumor biomarkers in sEV NanoPoms compared with ultracentrifugation or other bead isolation approaches (Figure 4). A few miRNAs and protein cancer biomarkers highly enriched in urinary sEVs were identified by this method for the first time.

SERS coding nanoprobes functionalized with bivalent cholesterol-labeled DNA strands were used for highly sensitive quantification of sEVs. The proposed approach enabled sEV detection as low as 27 vesicles per μL [71]. The simultaneous detection of SKBR3, T84 and LNCaP sEVs with the limit of detection from tens to hundreds of vesicles per microliter using aptamer-conjugated SERS-encoded probes on magnetically captured magnetic beads was demonstrated by Z. Wang [72].

## 5. Integrated Technologies

**Figure 5:**
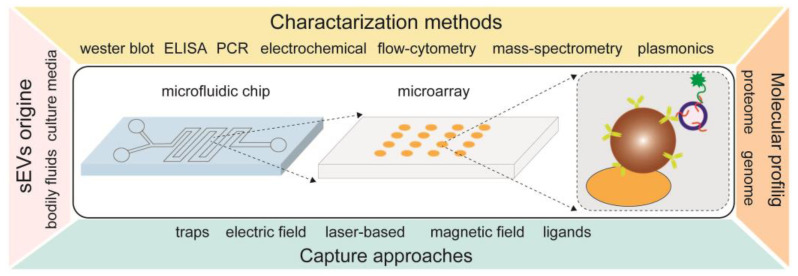
An overview of integrated technologies with bead-assisted platforms for isolation, quantification and molecular profiling of sEVs.

In addition to various types of particles being used as stand-alone tools for the isolation of sEVs, other groups demonstrated technologies where the particles are integrated onto a substrate for sEV capture and analysis. Figure 5 gives an overview of using bead-assisted platforms for the isolation of sEVs in combination with microfluidics and microarrays. In such combinations, various capture approaches can be applied, such as physical trapping, ligands, electric and magnetic fields and laser light. Moreover, once the sEVs are trapped, the physical and biochemical characteristics of sEVs can be analyzed by different methods, such as ELISA, mass spectrometry, flow cytometry, plasmonic-based and others.

Zhao Z. et al. developed a microfluidic chip (ExoSearch) from PDMS with an integrated 2 mm magnet disk and magnetic beads coated with capture antibodies for trapping sEVs [40]. This approach allowed the group to quantitate sEVs obtained from ovarian cancer patient plasma and healthy controls and perform multiplexed marker detection with the LOD of 7.5 × 10^5^ vesicles per mL. By using ExoSearch, it was shown that ovarian cancer patients overexpressed the CA-125, EpCAM, CD9, CD81 and CD63 markers. Subsequently, the group designed a microfluidic chip that, in addition to isolation of sEVs subpopulations, allows to lyse them and perform multiparametric analysis of intravesicular biomarkers [41]. Niu F. et al. introduced an S-shaped micromixer with an embedded baffle that allowed the effective capture of subpopulations of sEVs by the immunomagnetic bead. [73]. The fabricated chip was allowed to perform a complete isolation of a subpopulation of sEVs. The flow rate through the microfluidic channels was set to be 20 μL/min, with the total time that was required for the isolation of 150 μL serum samples being ~50 min. The authors used Western blot to verify the ability of the platform to isolate CD63 + sEVs. Another type of a microfluidic chip in the form of a microarray composed of channels that are separated by pillars was presented by Bai Y. and co-workers [74]. Such geometry with an optimized flow rate allows single beads with sizes slightly larger than the micropillar gap length to become trapped. If the gap is already occupied, the flow resistance of the gap would increase and cause the beads to bypass this site and end up in empty gaps. Commercial microbeads conjugated with anti-CD9 antibodies along with quantum dots (QD) for carcinoembryonic antigen (CEA), fragments of cytokeratin 19 and pro-gastrin-releasing peptide (Pro-GRP) were used to identify and fluorescently label sEVs isolated from lung adenocarcinoma (A549), lung squamous carcinoma (H226), small cell lung cancer (H446), human umbilical vein endothelial (HUVEC) cells and plasma from 10 patients who had not undergone primary surgical resection of lung cancer and 10 healthy controls. The group observed increased marker expression levels in lung cancer patients compared to controls, although it did not find a distinct difference in expression levels in different types of lung cancer. However, they were able to differentiate sEVs obtained from different lung cancer cell lines by using the presented method. Chen W. et al. produced a microfluidic device with multiple layers containing a sealing layer, chamber layer, channel layer and a magnet positioned between the cover and mounting plates [42]. The procedure of the lab-on-a-chip first started with the immunomagnetic beads with captured sEVs flowing through a channel where a magnet was present to capture them for further fluorescent labeling. The tetramethylbenzidine (TMB) substrate, which was added next under the catalysis of HRP, produces a soluble product causing a color change and allows the use of a spectrophotometer for optical density measurements. To evaluate the device’s applicability for clinical use, the group compared EpCAM expression in 10 breast cancer patients compared to 10 healthy controls. It was found that EpCAM expression evaluated by the microfluidic chip allows differentiating of breast cancer patients from control patients with 90% sensitivity and >95% specificity. Kabe Y. et al. introduced an ExoCounter for the quantification of bound sEVs by labeling them with antibody-conjugated magnetic beads [43]. ExoCounter allowed us to find high expression of CD9+/HER2+ sEVs in sera of breast and ovarian cancer patients compared to healthy donors. The LODs of ExoCounter were found to be 1.16 ng protein for sEVs purified from the cell culture medium and 0.39 ug for serum samples, which were significantly lower than counterparts such as ELISA and flow cytometry. Lin A. et al. fabricated chips where ferromagnetic metals were electro-deposited into self-assembled microlattice, which allowed them to produce billions of nanomagnetic traps that allowed to enrich magnetically labeled sEVs and obtain a purified sample for further characterization [75]. To capture subpopulations of sEVs the group used commercial anti-biotin magnetic nanoparticles that target sEV markers. Such magnetic nanoparticles with bound sEVs were trapped by a face-centered cubic immunomagnetic sorter (FIS). The group has shown that, compared with the commonly used methods for the isolation of sEVs, such as UC and precipitation, their approach allows the capture and purification of subpopulations of sEVs with the possibility of lysing them for further RNA extraction and qPCR. The total RNA yield of FIS was also shown to surpass the UC isolation approach. Jeong S. et al. developed and presented an integrated magnetic-electrochemical exosome (iMEX) platform that allows to capture of subpopulations of sEVs by magnetic beads containing capture antibodies and labeling them with target antibodies and horseradish peroxidase (HRP) for electrochemical detection by using chromogenic electron mediators, which generate an electrical current when HRP is present [44]. The group first performed testing of the system by using EVs isolated from cell lines and found a strong correlation between the expression of markers on the cell and sEV membranes. The iMEX platform was then tested by directly using 80 μL plasma obtained from 11 ovarian cancer patients and 5 healthy controls. It was found that expression levels of EpCAM and CD24 of CD63+ sEVs were significantly higher in ovarian cancer patients compared with healthy controls. The group also has shown the applicability of the developed method to monitor the dynamics of marker expression before and after drug treatment. Wei F. et al. presented an electric field-induced release and measurement (EFIRM) system to eventually detect the subpopulation of sEVs and their RNA after release by applying a non-uniform electrical field [45]. Magnets underneath the electrochemical sensor were used to capture the sEVs–magnetic bead complexes. Once fixed by the magnet, the csw E-field was applied to release GAPDH mRNA from captured sEVs. The mRNA was hybridized with oligonucleotide capture and detector probes, followed by adding an anti-fluorescein antibody conjugated to HRP for amperometric measurements. The group showed the ability to simultaneously measure sEVs CD63 surface proteins and detect harbored mRNA. It was shown that tumor-derived sEVs could be detected in saliva in addition to blood. Rho J. et al. developed a prototype micro-nuclear magnetic resonance (uNMR) system that allows to isolate and quantify microvesicles (MVs) in packed red blood cell (pRBC) units and label their subpopulations with target-specific magnetic nanoparticles [46]. In a recent study, Wang S. et al. developed and tested a rapid Cu nanoshell-enhanced immunoassay (Cu-NEI) where in situ Cu growth allows to significantly enhance NP-induced signal intensity [76]. The group first tested and compared AuR with AuS enhancement by Cu nanoshell growth. They found that Cu nanoshell growth significantly enhances plasmonic scattering intensity with AuR@Cu signal outperforming AuS@Cu hence AuR@Cu was used for experiments thereof. Slides having multiple wells conjugated with protein A/G were conjugated with a CD81 antibody. Pre-isolated sEVs or serum samples were added and labeled with biotinylated antibodies to CD63 or LAM, followed by avidin-conjugated AuR NPs. Cu-NEI has shown a greater overall pediatric TB diagnostic performance, including sensitivity (76%) and specificity (100%), compared with Mtb culture, Xpert, urinary LAM and AuR (without Cu nanoshell growth) assay methods. The Cu-NEI method was also shown to have LOD down to 39 sEVs/uL, significantly better than ELISA (5187 sEVs/uL).

It can be seen that the recently presented coupling of particles with other components, such as microfluidics, allows the creation of new technologies that, in the near future, provide the potential to achieve high sensitivity and specificity and become routinely used in clinics.

## 6. Conclusions

sEV isolation from biological fluids still remains a challenge due to their biophysical properties and the complexity of biofluids where sEVs reside. Although numerous approaches for sEV extraction and characterization were previously presented and compared, bead-assisted technology was the main focus in the review presented here to highlight the advantages and potential that outweigh other methods. The advantages include the versatility as to how beads can be manipulated and used to achieve the end goal, the requirement of only basic tools (e.g., centrifuge and/or magnet) needed throughout the protocols and compatibility with commonly used instrumentation such as flow cytometry that can be used for final molecular profiling. Beads that were previously presented and used for sEV capture were divided into magnetic or nonmagnetic types and further subcategorized into specific and nonspecific. In previous work, it was shown that beads could not only be used for the characterization of preisolated/purified sEVs but also for isolation directly from biological fluids such as blood plasma, serum and saliva. In addition, we presented recent integrated technologies where beads were coupled with systems such as microfluidics to minimize the required sample volume and time needed for sEV analysis. We anticipate that, in the near future, bead-assisted technologies will become utilized in clinical laboratories to perform routine testing for diagnostics and treatment monitoring. However, despite recent success in the extraction and characterization of sEVs by bead-assisted technology, some problems need to be addressed to enable the effective collection of target sEVs from real patient samples. The following factors will determine the efficiency of isolation of sEVs and should be taken into account: (i) the source and characteristics of sEVs; (ii) the characteristic of the antibody–antigen interaction; (iii) the nature and structure of the target molecule; and (iv) the ratio and concentration of the beads and target molecules. In addition, the beads should be biologically inert to prevent the binding of lipoproteins albumin, other proteins and their aggregates to the bead surface. Moreover, the complete isolation of sEVs from other EV subsets is yet another valuable problem that should be resolved.

## Figures and Tables

**Table 1:** Classification of extracellular vesicles.

	Small Extracellular Vesicles (sEVs)	Microvesicles (MVs)	Apoptotic Bodies (ABs)
**Size, nm**	30–150	100–1000	100–5000
**Biogenesis**	Inward budding of endosomal membranes	Outward budding of plasma membrane	Cell apoptosis
**Characteristic markers**	ALIX, TSG01, tetraspanins (CD9, CD63 and CD81), GM1 gangliosides and transferrin receptors, cholesterol, ceramide and sphingomyelin	Cholesterol, sphingomyelin, ceramide, CD40 ligand, ADP-ribosylation factor 6, integrins and flotillins	Annexin V, C3b, thrombospondin, histones and DNA fragments

## Data Availability

The data presented in this study are available upon request from the corresponding author.
